# Functional Foods: A Promising Strategy for Restoring Gut Microbiota Diversity Impacted by SARS-CoV-2 Variants

**DOI:** 10.3390/nu15112631

**Published:** 2023-06-05

**Authors:** Antara Banerjee, Indumathi Somasundaram, Diptimayee Das, Samatha Jain Manoj, Husaina Banu, Pavane Mitta Suresh, Sujay Paul, Atil Bisgin, Hong Zhang, Xiao-Feng Sun, Asim K. Duttaroy, Surajit Pathak

**Affiliations:** 1 Faculty of Allied Health Sciences, Chettinad Hospital and Research Institute (CHRI), Chettinad Academy of Research and Education (CARE), Kelambakkam, Chennai 603103, Tamil Nadu, India; antara.banerjee27@gmail.com (A.B.); dasdiptimayee04@gmail.com (D.D.); divyaacool24@gmail.com (S.J.M.); husnalateef250@gmail.com (H.B.); pavanemitta19@gmail.com (P.M.S.); drsurajitpathak@care.edu.in (S.P.); 2 Department of Biotechnology Engineering, Kolhapur Institute of Technology’s College of Engineering, Kolhapur 416012, Maharashtra, India; drindumathisomasundaram@gmail.com; 3 School of Engineering and Sciences, Tecnologico de Monterrey, Campus Queretaro, San Pablo 76130, Mexico; spaul@tec.mx; 4 Department of Medical Genetics, Medical Faculty, Cukurova University, Adana 01250, Turkey; abisgin@yahoo.com; 5 Department of Medical Sciences, School of Medicine, Orebro University, SE-701 82 Orebro, Sweden; hong.zhang@oru.se; 6 Division of Ocology, Department of Biomedical and Clinical Sciences, Linkoping University, SE-581 83 Linkoping, Sweden; 7 Department of Nutrition, Institute of Basic Medical Sciences, Faculty of Medicine, University of Oslo, 0313 Oslo, Norway

**Keywords:** COVID-19, SARS-CoV-2, gut microbiota, concern variants, ACE2 receptor, functional foods, gut microbiota metabolites

## Abstract

Natural herbs and functional foods contain bioactive molecules capable of augmenting the immune system and mediating anti-viral functions. Functional foods, such as prebiotics, probiotics, and dietary fibers, have been shown to have positive effects on gut microbiota diversity and immune function. The use of functional foods has been linked to enhanced immunity, regeneration, improved cognitive function, maintenance of gut microbiota, and significant improvement in overall health. The gut microbiota plays a critical role in maintaining overall health and immune function, and disruptions to its balance have been linked to various health problems. SARS-CoV-2 infection has been shown to affect gut microbiota diversity, and the emergence of variants poses new challenges to combat the virus. SARS-CoV-2 recognizes and infects human cells through ACE2 receptors prevalent in lung and gut epithelial cells. Humans are prone to SARS-CoV-2 infection because their respiratory and gastrointestinal tracts are rich in microbial diversity and contain high levels of ACE2 and TMPRSS2. This review article explores the potential use of functional foods in mitigating the impact of SARS-CoV-2 variants on gut microbiota diversity, and the potential use of functional foods as a strategy to combat these effects.

## 1. Introduction

Since ancient times, functional or therapeutic foods have been used to treat and prevent many ailments. Many plants and herbs contain bioactive compounds, such as tannins, polyphenols, flavonoids, and terpenoids, among others, known to possess therapeutic or preventive properties [1]. Additionally, using probiotics as food supplements can help fight infections and support the body’s natural defense mechanisms. Preventative measures against COVID-19 have gained popularity, and various functional foods effectively strengthen the immune response to fight infection. The nutritional status of an individual is a key factor governing the extent of severity of SARS-CoV-2 infection. Thus, supplementation with vitamins and minerals is helpful in prevention and treatment [2]. Several functional foods of therapeutic value are beneficial in this regard. Several vitamins, such as vitamins C, D, and E, have been shown to promote an immune response against viral infections [3].

Furthermore, they function as potent antioxidants and help to reduce levels of Reactive Oxygen Species (ROS) and cellular stress. Minerals in the form of micro-minerals or macro-minerals are essential for many biological functions [4]. Supplementation of some minerals, such as zinc, has an immune-protective effect as it promotes autophagy, cytokine production, anti-viral function, and enhancement of anti-viral drugs’ efficacy. Apart from functional foods, plant-derived small molecules or herbal formulations are found to be effective in reducing the detrimental effects of COVID-19 infection. Many of these have been a part of traditional medicine in various cultures. The isolation and characterization of their bioactive molecules has proven their protective effect in various clinical conditions. Notably, incorporating functional foods and herbal formulations as a therapeutic strategy against COVID-19 infection has benefits in terms of prevention through strengthening the immune system and diminishing the extent of infection.

The emergence of the SARS-CoV-2 virus, which caused the COVID-19 pandemic, has severely impacted public health and the world economy [5]. As the virus mutates, it produces different variants that vary in their ability to spread and the severity of the infections they cause [6,7]. Unlike the original strain, these variants are also affecting younger individuals. Additionally, the SARS-CoV-2 virus is more contagious than the SARS-CoV virus [8]. The genetic makeup of SARS-CoV-2 has undergone considerable changes, leading to different variations compared to the first identified genetic sequence of Wuhan-Hu1 [9]. The identification and nomenclature of genetic sequence mutations are carried out using Phylogenetic Assignment of Named Global Outbreak (PANGO). The nomenclature employs Greek letters, i.e., alpha, beta, delta, etc., to denote variants and a series of letters and numbers to assign lineages, i.e., BA.1.1, B.A.2, etc. [10]. Mutations in the SARS-CoV-2 receptor-binding domain have given rise to new SARS-CoV-2 variants, including Alpha (B.1.1.7), Beta (B.1.351), Gamma (P.1), Delta (B.1.617.2), Cluster 5, B.1.1.207, B.1429/B.427, B.1.525, Omicron (B.1.1.529), Mu (B.1.621), and LAMBDA (C.37).

Based on their impact on public health, the emerging SARS-CoV-2 variants have been categorized by the World Health Organization (WHO) into three groups: variants of concern (VOC), variants of interest (VOI), and variants under monitoring (VUM) [11]. The mutations in SARS-CoV-2 can be either deleterious or neutral, resulting in resistance to existing treatment strategies and showing either higher infectivity or no apparent effect, respectively [12]. The viral phenotype is modified via a small set of mutations conferring an advantage regarding their pathogenicity and transmissibility. The antigenicity of mutant SARS-CoV-2 modulates immune recognition properties, leading to variants’ escape from the host immune response. SARS-CoV-2 variants with higher accumulation of such mutations have shown enhanced transmissibility and are categorized under VOC. It has been shown that nearly 90% of plasma antibodies act against the spike receptor-binding domain (RBD) [13].

Patients infected with COVID-19 commonly display respiratory symptoms, such as fever, fatigue, cough, shortness of breathe, and abnormal chest X-ray results. Additionally, many COVID-19 patients experience gastrointestinal issues, such as nausea or vomiting, diarrhea, abdominal pain, and loss of appetite [14]. Studies have found that the balance of microbiota in the respiratory and gastrointestinal systems of hospitalized COVID-19 patients is disturbed. This disruption may make patients more susceptible to secondary respiratory and gastrointestinal tract infections, contributing to the severity of COVID-19 [15]. Therefore, the microbiota may play a crucial role in SARS-CoV-2 infection. The human body contains ten times more bacterial cells in its microbiota than human tissue cells and a hundred times more bacterial cells than human genes. These bacteria inhabit all areas of the human body, including the respiratory and gastrointestinal tracts, and are selectively allowed to colonize. The microbiota performs several vital functions in the human body, including decomposing indigestible proteins and carbohydrates, nutrient absorption and digestion, vitamin production, and regulating host immunity. The microbiota is also linked to various diseases and significantly impacts human health [16,17,18].

Managing COVID-19 infection is a significant challenge in light of its serious implications on systemic functions and its adverse effects on immunity. In this regard, natural herbs and functional foods provide an excellent way to minimize the effects of the SARS-CoV-2 virus through various mechanisms, such as anti-viral functions, immune-boosting functions, antioxidant functions, and maintaining gastrointestinal microbiota homeostasis.

There have been suggestions that probiotics could help reduce the spread of COVID-19, but there is currently a lack of evidence to support this [19]. However, this also highlights the need for further research and a better understanding of the microbiome. One major challenge is the potential for misinterpretation of data and unrealistic expectations for how quickly complex research can be translated into practical applications by society, scientists, and healthcare professionals.

So far, the use of probiotics in relation to COVID-19 has mainly been supported by indirect evidence [20]. The current understanding is that the gut microbiome, which refers to the combined genetic material of the various microorganisms in the human gastrointestinal tract, can regulate and be regulated by viruses entering the body, leading to positive or adverse effects. Furthermore, certain beneficial bacteria in the gut can help defend against potential pathogens by interacting with human cells and promoting healthy immune function [21]. This suggests that dietary microbes, such as probiotics or prebiotics, could positively impact immune function during infections [22].

This review provides an overview of recent research on how COVID-19 affects the gut microbiota, including the specific bacteria involved and their impact on immunity and disease severity. Additionally, it discusses what is currently known about the potential for probiotics, prebiotics, and nutritional interventions to reduce susceptibility to COVID-19. Finally, the review examines several studies that have used plant-based probiotics to target coronavirus, and it considers implications for future research design and the potential role of precision nutrition in COVID-19.

## 2. What Qualifies a Food as “Functional”?

Functional foods result from advancements in nutritional science that emerged during the same period as the baby boomer generation. Experiences of food rationing during wartime highlighted the importance of population-based strategies to prevent dietary deficiencies [23]. To address this, free school milk was introduced, and vitamins and minerals were added to certain foods to ensure people met their recommended intake. This led to the acceptance of fortified breakfast cereals as a healthy part of the lifestyle of a new generation of Australians. Thus, a new era of “functional foods” began, which refers to foods that provide sustenance and that positively impact consumers’ health.

Functional food science is the result of collaboration between different fields of science and the needs of the general public. It combines food science, nutrition, and medical principles to create foods that straddle the boundary between food and pharmaceuticals. Researchers in this field study food’s various components and potential health benefits. Functional food scientists utilize biomarkers or “indicators” present in the body to track changes in health and the body’s ability to maintain equilibrium. This technique helps assess the effects of functional foods on health to determine the appropriate and safe dosages of these foods. Functional foods can be classified based on their intended health benefits or the ingredients that provide those benefits [24]. These classifications are not mutually exclusive; many functional foods may fit into multiple categories. Here are some common classifications:

Nutraceuticals: Foods or food products that contain bioactive compounds (such as vitamins, minerals, or antioxidants) that have medicinal properties.

Fortified/Enriched Foods: Foods that have additional nutrients added to them to increase their nutritional value (such as vitamin D-fortified milk or iron-enriched cereal).

Probiotics: Foods that contain live beneficial bacteria that help improve gut health and boost the immune system.

Prebiotics: Foods that contain non-digestible fibers that promote the growth of beneficial gut bacteria.

Dietary Supplements: Products that contain one or more ingredients (such as vitamins, minerals, or herbs) intended to supplement the diet.

Functional Beverages: Beverages that contain added nutrients, such as vitamins or minerals, or bioactive compounds, such as antioxidants.

Whole Foods: Certain foods, such as fruits, vegetables, nuts, and whole grains, that have inherent health benefits and can be considered functional foods when consumed as part of a healthy diet.

In the 1980s, functional food emerged in Japan as scientists provided evidence of their products’ beneficial effects on health [25]. Upon approval, these foods were given a special label: FOSHU (Food for Specific Health Uses). Other countries and scientific organizations attempted to develop their own definitions of functional foods, which resulted in high sales but also led to confusion among consumers about what the term “functional food” means. The Functional Food Center (FFC) has defined functional food as natural or processed foods that contain biologically active compounds, whether known or unknown [26]. When consumed as defined and effective, these foods have clinically proven and documented health benefits for preventing, managing, or treating chronic diseases. This definition is distinctive in recognizing the importance of “bioactive compounds”, which are biochemical molecules that enhance health through physiological mechanisms.

Moreover, this definition emphasizes that bioactive compounds must be consumed in non-toxic amounts, as there are upper limits beyond which they can become hazardous. The main goal of the FFC is to establish a uniform definition of functional foods that will enhance the credibility of functional food research. For instance, functional food might protect against infection, prevent supplement deficiency, advance appropriate health development and improvement, and prevent and fight microbial infection and gastric ulcers. Additionally, it is essential for the center’s aims to promote the commercialization of functional foods, enhance global collaboration and communication, and, ultimately, improve the health of the population.

## 3. SARS-CoV-2 Infection in the Gastrointestinal Tract

The binding of SARS-CoV-2 to its receptor, human angiotensin-converting enzyme 2 (ACE2), is a vital stage in the infection’s progression. SARS-CoV-2 binding depends on interactions between ACE2 and the receptor-binding domain (RBD) in the virus’s spike (S) protein. ACE2 is present in several body tissues, such as the cardiovascular system, kidneys, testicles, and the gastrointestinal tract, including the colon, cecum, duodenum, ileum, and jejunum. This implies that SARS-CoV-2 can affect the gastrointestinal tract, leading to irregular intestinal secretion, malabsorption, and other gastrointestinal symptoms [16,17]. The gut virome can affect the host’s health or disease state by influencing the immunophenotype rather than acting as a pathogen. The detection of SARS-CoV-2 RNA in the stool of COVID-19 patients suggests that the virus can spread through the fecal–oral route [18]. The virus has been found in various parts of the gastrointestinal tract, and the nucleocapsid protein has been found in the stomach, rectum, and duodenum glandular epithelial cells. In some cases, viral RNA was still detectable in feces even after negative respiratory tests, indicating that the virus was still replicating in the gastrointestinal tract, and fecal–oral transmission could continue even after respiratory clearance [27,28,29,30,31].

### 3.1. Gastrointestinal Complications in COVID-19

**Figure 1:**
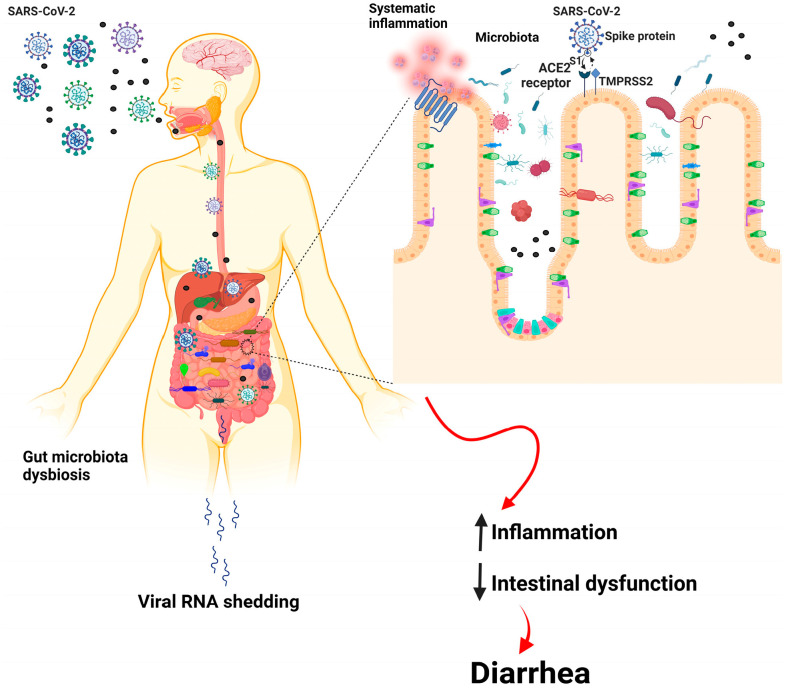
Basic pathway of the gut–lung axis in COVID-19-infected individuals.

COVID-19 patients have been found to have bowel wall thickening with hyperemia, mesenteric thickening, fluid-filled large bowel, and, sometimes, ischemia and pneumatosis, as shown by imaging techniques [32]. In addition to fever and respiratory symptoms, COVID-19 patients commonly experience digestive symptoms, such as nausea, anorexia, vomiting, and diarrhea. Some COVID-19 patients experience only digestive symptoms, while others may have these before developing respiratory symptoms. Systematic reviews and meta-analyses have shown that gastrointestinal symptoms occur in 2–21% of COVID-19 patients, with the most common being diarrhea (8–17%), nausea or vomiting (4–20%), and loss of appetite (2–21%) [33]. Dysregulation of intestinal ion transporters due to SARS-CoV-2 infection may cause infectious diarrhea and malabsorption, leading to inflammation and gastrointestinal symptoms. Patients infected with SARS-CoV-2 commonly experience diarrhea along with fever and cough. Animal studies have shown that changes in the ACE2 enzyme can lead to susceptibility to diarrhea and inflammation in the colon. In a retrospective analysis of 84 SARS-CoV-2 patients in Wuhan Union Hospital, viral RNA was detected in the fecal samples of all patients with diarrhea. A statistical analysis of 35 studies showed that 15% of COVID-19 patients experienced digestive symptoms, such as diarrhea, vomiting, nausea, and lack of appetite [34]. Gut dysbiosis is thought to be the primary cause of diarrhea in COVID-19 patients. However, further research is needed to understand the mechanisms underlying viral infections causing diarrhea and the relationship between respiratory and gastrointestinal symptoms [35] (Figure 1).

### 3.2. Alteration of Gut Microbiota upon SARS-CoV-2 Infection

The human gastrointestinal tract and respiratory tract host microbiota is a community of microorganisms numbering in the trillions, as demonstrated in Table 1. This microbiota is crucial for maintaining metabolic homeostasis and facilitating immune maturation [36]. A “healthy” microbiome interacts functionally and continuously with the host organism through surface antigens and microbial metabolites. This interaction provides necessary molecular signals to fine-tune immune responses in various body parts, including the respiratory system, and protect against disease [37]. The gut microbiota can influence immune cells’ maturation, development, and function, and the activation of peripheral immune cells, including cellular and humoral immunity [38]. Disrupting gut barrier integrity and releasing proinflammatory cytokines into the circulatory system activate innate and adaptive immune cells, leading to systemic inflammation. The gut microbiota is disrupted by entering inflammatory cells, such as neutrophils and lymphocytes, into the intestinal mucosa [39].

**Figure 2:**
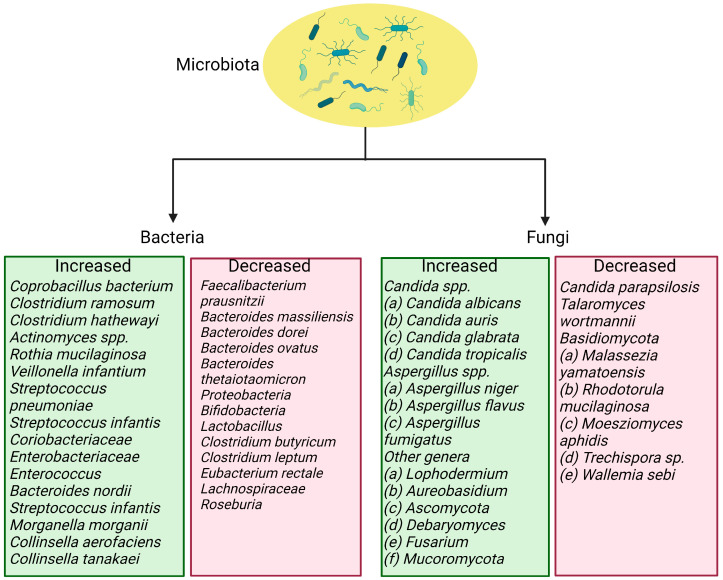
Bacteria and fungi that are increased and reduced in SARS-CoV-2 infection.

The composition of gut microbiota changes after viral infection. The severity of the disease is linked to an increase in the concentration of inflammatory cytokines and blood markers, such as C-reactive protein, lactate dehydrogenase, aspartate aminotransferase, and γ-glutamyl transferase [40]. A viral infection can cause intestinal wall permeability and malabsorption by enterocytes. Patients infected with SARS-CoV-2 have elevated fecal calprotectin levels, indicating intestinal inflammation [41]. A recent systematic review showed that COVID-19 patients had a depletion in the genera *Ruminococcus*, *Alistipes*, *Eubacterium*, *Bifidobacterium*, *Faecalibacterium*, *Roseburia*, *Fusicathenibacter*, and *Blautia*, as well as an enrichment of *Eggerthella*, *Bacteroides*, *Actinomyces*, *Clostridium*, *Streptococcus*, *Rothia*, and *Collin-sella,* as shown in Figure 2 [42].

Respiratory dysbiosis can also influence the digestive tract through immune control, and the composition and function of the gut microbiome can affect the respiratory tract through the underlying mucosal immune system [43]. In patients with COVID-19, changes in the gut microbiome are associated with disease severity and poor prognosis. Specifically, there are increases in certain bacteria, such as *Bacteroides*, *Parabacteria*, and *Clostridium*, and reductions in others, such as *Roseburia*, *Eubacterium*, and *Faecalibacterium*. The *Ascomycota* phylum indicates higher relative abundance and decreased diversity and richness in the fungal gut microbiota of severe/critical COVID-19 patients [44,45]. Additionally, patients with severe SARS-CoV-2 infection have significantly lower bacterial diversity and lower relative abundances of certain beneficial bacteria in the gut microbiome. Therefore, correcting the gut microbiota during the COVID-19 pandemic may help improve population immunity and protect public health [46].

The severity of COVID-19 is linked to the relative abundance of certain bacteria in the gut microbiome. While *Faecalibacterium prausnitzii*, which contains anti-inflammatory characteristics, exhibits an inverse association with disease severity, *Coprobacillus*, *Clostridium ramosum*, and *Clostridium hathewayi* show positive correlations with disease severity [47]. Moreover, fecal samples with high SARS-CoV-2 infectivity contain more opportunistic pathogenic bacteria (such as *Collinsella* species and *Morganella morganii*) and fewer bacteria that produce short-chain fatty acids (such as Parabacteroides merdae and *Lachnospiraceae* bacterium) compared to samples with low SARS-CoV-2 viral infectivity [41].

*F. prausnitzii* is a type of bacteria that lives in the human body without causing harm and has been found to have anti-inflammatory effects in clinical studies [48,49]. By secreting bioactive chemicals, it can prevent intestinal epithelial cells from activating nuclear factor-kB and producing IL-8. Additionally, *F. prausnitzii* can stimulate the secretion of IL-10, which inhibits the production of proinflammatory cytokines and enhances the suppressive activity of Tregs in the mucosa [50]. This bacterium is an important contributor to the body’s defense mechanisms, and its depletion has been observed in COVID-19 patients. During hospitalization, the abundance of certain intestinal microorganisms showed a reverse trend compared to the load of SARS-CoV-2 in fecal samples of patients. *Coprobacillus* was found to upregulate the expression of ACE2, while *Bacteroides thetaiotaomicron*, *Bacteroides dorei*, and *Bacteroides massiliensis* were found to downregulate ACE2 expression in the guts of patients. Overall, these findings suggest that harmful bacteria and the absence of beneficial bacteria in the gut are closely related to the severity of COVID-19 [51,52].

### 3.3. Loss of Gut Microbial Diversity and Associated Risk Factors in COVID-19 Patients

Dysbiosis occurs when the gut bacterial balance is interrupted. An imbalance in bacterial composition, changes in bacterial metabolic activity, or changes in bacterial distribution within the gut are symptoms of dysbiosis. Dysbiosis may be classified into three types: (1) loss of beneficial bacteria, (2) overgrowth of potentially harmful bacteria, and (3) loss of overall bacterial diversity. Gut microbial diversity often decreases with age, possibly due to changes in physiology, food, medicine, and lifestyle. Reduced diversity has been related to various chronic diseases, including obesity, type 2 diabetes, and COVID-19. Risk factors and comorbidities associated with COVID-19 include dementia, Alzheimer’s disease, male gender, smoking, hypertension, and asthma [52]. All these risk factors and comorbidities are associated with changes in the gut microbiome. These changes lead to alterations in the immune system and increase susceptibility to suffering from the adverse effects of SARS-CoV-2 [53]. Microbial diversity reduces as aging occurs, which causes severe inflammatory conditions. As such, SARS-CoV-2 infection seems more perilous in older people and in people with diseased conditions. Although the evolution of gut microbiota during human life is similar to older people’s gut microbiota, infant gut microbiota also shows decreased diversity [54]. The microbiota in infants remains unstable in the initial years until it changes to adult microbiota [55]. In a healthy individual, the gut microbiota is believed to be stable throughout their life, and its diversity remains high [56]. In old age or in diseased individuals, gut microbiome diversity diminishes, and gut dysbiosis rises, which is associated with mental impairment, inflammation, and depression [57]. In older adults, it was also identified that the gut microbiota contains reduced levels of specific bacterial groups. Nonetheless, bacterial groups, such as *Lactobacillus*, *Bacteroides*, and *Clostridium*, show recurrent alterations [58].

It has been stated that more COVID-19 fatalities are associated with older individuals due to the following reasons: (1) lessened microbial diversity, (2) weak immune system, and (3) systemic inflammation. Notably, gut microbiota diversity decreases with aging, so COVID-19 has become a fatal infection in most elderly patients [17]. Besides older age, obesity is also correlated with changes in intestinal microbiota, which serves as one of the risk factors for the severity of SARS-CoV-2 infection. Intriguingly, 25% of the patients who died from COVID-19 infection in the United States were found to be obese [59]. Adipose tissue serves an essential role in systemic immune activation and widespread COVID-19. Adipocytes show a heightened level of ACE2 expression in obese individuals, and eliminating existing inflamed adipose tissue reduces viral entry and systemic viral spread [60].

Moreover, adipose tissue shows irregulated lymphoid and myeloid responses in individuals with obesity, which is correlated with improper regulation of cytokine profiles [61]. Furthermore, obese individuals show increased chemerin levels, a proinflammatory cytokine, and leukotriene, worsening the cytokine storm risk [62]. Additionally, COVID-19 patients with diabetes have a high chance of facing severe complications accredited to gut metabolite dysfunction and systemic inflammation [63]. Likewise, SARS-CoV-2-infected patients with cardiovascular diseases, hypertension, nervous disorders, and other diseases may experience worsened complications of COVID-19 due to an imbalance in the intestinal flora and reduced microbial diversity [64]. Pre-existing diseases, such as cancer, cardiovascular disorders, lung problems, hyperglycemia, and diabetes, increase mortality risk during COVID-19 and influenza viral infections. These morbidities lead to gut dysbiosis, circadian dysregulation, and mitochondrial dysfunction [65]. There is proof that the microbiome in the gastrointestinal tract impacts the metabolic, digestive, and immune systems, host sleep, mental states, and circadian rhythms via the microbiome–gut–brain axis [66]. It was found that microorganisms and circadian genes are associated. Therefore, psychological status and good sleep duration and quality are essential to cope with the COVID-19 pandemic. Nevertheless, various drugs exert adverse effects by destroying the gut’s and lung’s microbiome integrity and worsening the individual’s potential to overcome COVID-19 infection and other viral infections and pathogenicity [67].

## 4. Therapies in the Management of Gut-Related Symptoms in COVID-19 Patients

**Figure 3:**
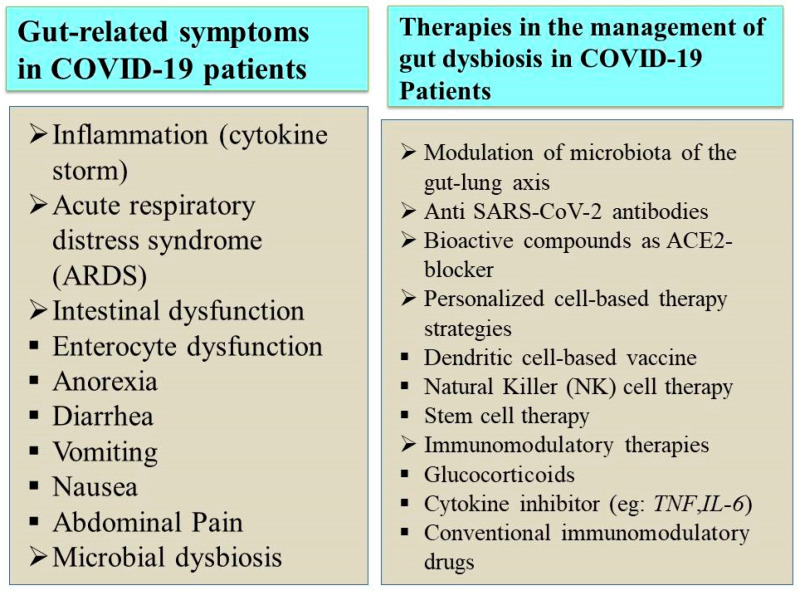
SARS-CoV-2 infection and its associated gut microbiome dysbiosis.

COVID-19 patients with gastrointestinal symptoms, such as diarrhea, vomiting, etc., cannot be cured entirely with antidiarrheal or antiemetic drugs. Hence, regular monitoring of potassium levels and sufficient rehydration solutions must be given to save patients’ lives [68]. Several therapeutic measures have been developed after the advent of COVID-19 based on various targets, as depicted in Figure 3. Interestingly, SARS-CoV-2 infection can be eliminated initially using novel therapeutic approaches, including activated epithelial receptors. The immunomodulation of the host’s innate immunity activates these epithelial receptors [69]. In addition, the intake of complex polysaccharides, proteins from plants, omega-3 fatty acids, polyphenols, and essential micronutrients is associated with increased beneficial bacterial quantity, stimulating SCFA production [70].

Probiotics are highly recommended to help alleviate gut-related symptoms, such as nausea and abdominal pain, and to increase gut microbial diversity [71]. In addition, the consumption of probiotics can help prevent severe COVID-19 clinical manifestations primarily related to the digestive tract [72]. Probiotics may have an especially large impact on the health of the gut microbiota of the elderly and individuals with several medical conditions [73].

### 4.1. Influence of Prebiotics and Probiotics on SARS-CoV-2 Infection

The role of prebiotics in regulating intestinal microbiota is widely recognized. Prebiotics refer to special fibers that contain fermenting ingredients necessary for gut microbes. These fibers selectively stimulate beneficial indigenous probiotic bacteria growth and/or activity. The use of prebiotics as a form of treatment has been linked to the improvement of gastrointestinal conditions, such as constipation and diarrhea, as well as non-gastrointestinal ailments, such as decreasing the chances of osteoporosis, atherosclerotic cardiovascular diseases linked with dyslipidemia, insulin resistance, obesity, and, conceivably, type-2 diabetes [74]. Studies conducted on mice indicated that inulin, a dietary fiber, protects against influenza A by affecting specific immune and T cells’ production. Traditional Chinese medicine has also been used to prevent, treat, and aid in the recovery from COVID-19, with a particular treatment called the “Lung Cleansing and Detoxifying Decoction” showing promise in relieving symptoms in 90% of patients after a 3-day course [75]. Prebiotics, a dietary fiber that selectively stimulates the growth of beneficial gut bacteria, include carbohydrate polymers, such as oligosaccharides and polysaccharides. Polysaccharides have been found to possess immunomodulatory properties, which are significant in enhancing cellular immunity and fighting viral infections [76]. The growth and function of probiotics are closely linked to prebiotics.

**Figure 4:**
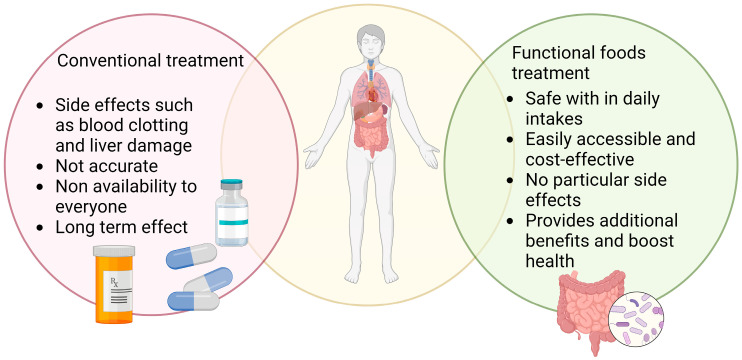
This representative image shows the influence of conventional treatment and functional food treatment on SARS-CoV-2 infection.

Gut microbes, including probiotics, break down different types of prebiotics, such as fructan, glucan, and arabinoxylan. This process produces short-chain fatty acids (SCFAs) that have been shown to regulate the host immune system. SCFAs affect the host immune response by activating G-protein-coupled receptors (GPCRs) through specific receptors with varying degrees of intensity [77]. T cells, macrophages, and dendritic cells’ ability to differentiate or carry out particular functions may be influenced by the butyric and propionic acids that the gut microbiota produces through prebiotic fermentation (Figure 4).

Initially, it was believed that the positive effects of probiotics, defined as live microorganisms that provide health benefits to the host when administered in sufficient amounts, were due to their ability to improve the balance of microbes in the intestine. However, recent evidence suggests that probiotics can also offer advantages by regulating the host’s immune system [78]. Probiotics have been demonstrated to influence the T cell subsets [79], stimulate the production of anti-microbial peptides by Paneth cells [80], and trigger Th17 cell differentiation in the small intestine [81]. To identify and design effective treatments, it is crucial to comprehend the basic immunological mechanisms that underlie the clinical effects of SARS-CoV-2 infection. Certain probiotics have been found to have anti-viral properties, including against other types of coronaviruses, according to laboratory research [82]. Researchers recently found that *Paenibacillus* bacteria naturally produce carboxypeptidases structurally and functionally, similarly to ACE2 [83]. Preventive intranasal administration of the probiotic *Lacticaseibacillus rhamnosus* GG was demonstrated to impact Toll-like receptor (TLR4) signaling in a neonatal mouse model of influenza infection [84]. Notably, LL-37, a human cathelicidin, is an anti-microbial peptide with broad-spectrum activity against viruses and bacteria [85] that can be effectively delivered by food-grade probiotics [86]. Using cathelicidin-expressing *Lactococcus lactis* as a probiotic delivery system may offer new opportunities for combatting SARS-CoV-2 infection through protective immunomodulatory effects. The WHO recently released new guidelines stating that mild to moderate COVID-19 patients should not receive antibiotic therapy or prophylaxis unless there is a suspected bacterial infection. However, using antibiotics in the early phase of COVID-19 can lead to a disrupted microbiota, increasing susceptibility to secondary infections. Therefore, using probiotics to reinforce the gut microbiota has been proposed as a possible way to reduce the risk of subsequent infections. However, additional clinical studies are required to determine the most appropriate strain(s), dose schedules, and timing of probiotic intervention for SARS-CoV-2 infection.

### 4.2. Probiotics Modulate Gut Microbiota

**Figure 5:**
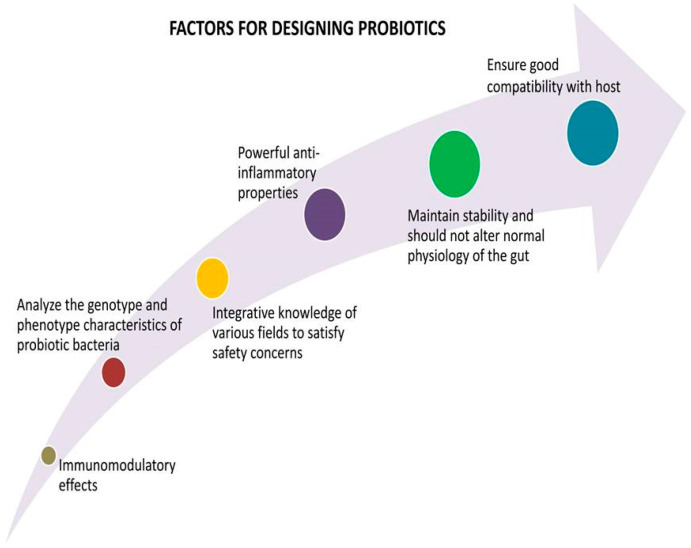
Strategies for designing successful probiotics.

The National Health Commission and the National Administration of Traditional Chinese Medicine advised incorporating a probiotic diet and edible products to treat patients with COVID-19 [87]. Probiotics are evolved from a live culture that enhances the balance of gut microbiota diversity to specific effects with the capability of immune-modulation effects [88,89]. Many desirable therapeutic aspects are considered for designing a probiotic product, as shown in Figure 5. Dairy foods are present-day common food carriers to supply probiotics. An analysis of the genotypic and phenotypic characteristics of bacteria is necessary to gain new insights into the selection of probiotic food products and the potential for bacterial selection as probiotics [90].

Besides this analysis, deeper study of host–gut microbial crosstalk is necessary for personalized treatment. The disclosure of metabolic activities of bacterial strains in the gut is the best way to determine the dynamics of the gut ecosystem [91,92]. Bacterial strains mostly belonging to the genera *Lactobacillus* and *Bifidobacterium* possess beneficial characteristics and act as potential sources for probiotics. Some of these strains showcase effective anti-inflammatory characteristics [93]. Probiotics are constituents of edible products that benefit the host when consumed properly and suitably. More probiotic effects are present in plant-based fibers, which promote beneficial bacteria, such as *Lactobacillus* and *Bifidobacterium* species [94,95]. At the same time, they reduce the growth of harmful pathogens, e.g., *Clostridium* species. Intake of fiber-rich foods reduces fatality risk from respiratory and infectious diseases by 40% [96]. Whole grains have sufficient intestinal microbiota composition, which decreases TNF-α (Tumour Necrosis Factor-α), C-reactive protein (CRP), and Interleukin-6 (IL-6) in the gut and circulation, which in turn reduces systemic inflammation and intestinal inflammation [97]. The fiber content of legumes, fruits, and vegetables fermented by intestinal flora and the beneficial metabolic compounds formed after fermentation have anti-inflammatory properties [98].

The SCFAs, such as butyrate, acetate, and propionate, formed by the microbes in the gut have anti-inflammation properties. SCFAs enhance the synthesis of anti-inflammatory cytokines and suppress the synthesis of proinflammatory cytokines by binding to the receptors of immune cells [99,100]. Nowadays, research has focused on investigating the correlation between gut bacterial molecular characteristics and their impacts on individual health conditions. Indeed, there are many significant challenges to elucidating the molecular bases of interaction-mediated systemic effects [65]. Priority should be given to safety concerns in manipulating bacterial strains and probiotic products for preventive and therapeutic interventions [101]. The probiotics are categorized by their mechanisms of action within COVID-19 patients and are listed in Table 2.

### 4.3. Natural Small Molecules and Functional Foods against COVID-19 Infection

Various herbal formulations derived from plants of therapeutic value have been tested for their efficacy against COVID-19 infection. Phytochemicals comprise different bioactive primary and secondary metabolites with well-known potent pharmaceutical properties. Natural compounds, such as quercetin, scutellarin, and myricetin, among others, are effective against coronavirus infection and have led to further studies [73,87,88,89].

*Echinacea purpurea* is an herbal medicine used to treat respiratory infections by Native Americans [90,91]. It consists of compounds, such as chicoric acid, caffeic acid, polysaccharides, and alkylamides, and is used in extracts, teas, sprays, and tinctures [92]. *Echinacea purpurea* has been reported to be effective against viral infections, especially enveloped viruses. Because SARS-CoV-2 is an enveloped virus, *Echinacea purpurea* can be useful as a therapeutic against COVID-19 infection.

Turmeric is well known for its medicinal properties due to the presence of bioactive compounds classified as curcuminoids, such as curcumin, demethoxycurcumin, and bisdemethoxycurcumin [93]. It has been reported to possess antioxidant, anti-inflammatory, and anti-cancer properties [94,95]. Curcumin is effective against COVID-19-induced anosmia (loss of smell) and ageusia (loss of taste). The possible mechanism for the action of curcumin in relieving anosmia and ageusia may involve binding to Mpro, a protease responsible for the production of proteins involved in viral replication. Furthermore, it can inhibit the complex formation between the SARS-CoV-2 spike protein and ACE2, thereby impeding viral entry [96].

Quinine alkaloids obtained from the bark of Cinchona trees have been used as anti-malarial drugs. Evidence gathered from several studies suggests their efficacy against viral infections, and they can be potent candidates for treating COVID-19 infections [97,102].

*Curcuma xanthorrhiza,* commonly known as Java turmeric, originates from Indonesia. It is a food additive and medicine due to its anti-microbial, anti-inflammatory, hyperglycemic, anti-hypertensive, anti-cancer, and anti-cancer nephron-protective properties [99,100,101,103,104]. The bioactive ingredients in *Xanthorrhizol* consist of curcuminoids, *Xanthorrhizol*, camphor, and volatile oils [105]. *Xanthorrhizol* has immunosuppressant properties and it can inhibit proinflammatory cytokines, which can be effective against COVID-19-associated cytokine release syndrome (CRS) [106]. However, clinical studies for its efficacy are not yet available, and it is suspected that immunosuppression can potentially cause more harms than benefits in the case of COVID-19 infection.

Polyphenolic compounds, such as flavonoids, tannins, and rosmarinic acids, are plant-derived bioactive molecules that have an aromatic ring with one or more hydroxyl groups. Polyphenols have been reported for their efficacy against diseases, such as cancer and neurodegenerative disorders, owing to their anti-inflammatory properties and ability to interact with multiple target proteins of etiological importance [107,108,109,110]. These compounds can interact with and inhibit proteins involved in viral replication, and they can be beneficial as a treatment strategy against COVID-19 infection [111,112,113,114].

Glycyrrhizin, a triterpene saponin derived from licorice root, is known to have therapeutic value due to its anti-inflammatory, anti-microbial, and antioxidant functions. It can bind to ACE2 and it has been reported to be effective in combining boswellic acid in treating COVID-19 [115].

Quercetin is a polyphenol of the flavone class of compounds known to possess antioxidant, anti-inflammatory, immuno-protective, and anti-viral properties. It is naturally present in various vegetables and fruits, such as onions, berries, apples, cilantro, etc. [116]. It can hinder the interactions of the SARS-CoV-2 spike protein with ACE2, thus inhibiting the entry of the virus into the host [117].

Alkaloids represent a large group of secondary metabolites mainly derived from flowering plants, fungi, and bacteria. The broad class of alkaloid compounds includes quinolines, purines, imidazoles, indoles, etc. [118,119]. Alkaloids exhibit multiple therapeutic properties in the form of antioxidant, anti-viral, anti-fungal, and anti-bacterial functions. Various studies demonstrate their action against COVID-19 infection as an individual drug molecule or in combination with other potential therapeutic molecules, such as caffeine, emetine, colchicine, etc. [119]. However, further studies are required to explore alkaloids’ role as a therapeutic molecule against COVID-19 infection.

Functional food comprises vital components, including multivitamins, which function as cofactors in various biological pathways and to ensure proper function [120]. Vitamin D plays a crucial role in immune functions. It is known to mediate the induction of anti-microbial peptides, such as cathelicidin, LL37, and defensins [121]. It also induces monocyte differentiation and antibody formation, promotes macrophage-mediated phagocytosis, and inhibits lymphocyte proliferation. Furthermore, it plays a role in anti-inflammation by reducing proinflammatory cytokine expression while enhancing anti-inflammatory mediators’ production by macrophages [122]. It has been reported that vitamin D decreases the expression of DPP4/CD26, a receptor involved in interaction with the SARS-CoV-2 spike protein. It also reduces sustained interferon-γ and the level of Interleukin-6, which helps improve pneumonia in COVID-19 patients [123].

Vitamin C is well known for its antioxidant functions and its role in supporting innate and adaptive immunity [124]. It can reduce susceptibility to infections and restores stress response functions. It also prevents the formation of neutrophil extracellular traps responsible for organ damage in COVID-19 patients. Vitamin C relieves the complications of thrombotic or thromboembolic diseases commonly observed in COVID-19 patients [125,126].

Minerals are required for various important biological functions, and their optimum quantity should be supplied in the diet or through supplements. Elements, such as zinc, iron, copper, and selenium, are required in micro-quantities, while sodium, potassium, phosphorous, and magnesium are needed in greater amounts [127,128]. Most trace elements function as co-factors in various enzymatic functions but act as direct modulators by binding to proteins [129]. Zinc is known to possess anti-viral action by causing hindrance to viral attachment. It has also been associated with immune functions by playing a role in the differentiation and development of immune cells. A deficiency of zinc is linked with hampered immune functions, such as reduction in antibody formation, low cytokine production, thymus atrophy, and reduced natural killer cell activity [130].

Nutraceuticals include food components known to have therapeutic actions against various ailments, such as diabetes, cancer, and neurological disorders [2]. These are derived from natural herbs, dietary supplements, and processed food. Incorporating nutraceutical products into the diet improves overall health and protects against chronic illness [131]. Ginseng is a traditional medicine that has immunomodulatory functions and anti-inflammatory action. It is beneficial in respiratory diseases and relieves the common cold and flu. However, no current evidence establishes its benefits for COVID-19 infection [132,133].

Omega-3 fatty acids have anti-inflammatory and immunomodulatory functions, which help reduce infection severity. They are generally required for cellular health and promote optimum cardiovascular, pulmonary, and endocrine functions [134,135].

## 5. Discussion and Conclusions

Although COVID-19 is primarily known for causing respiratory symptoms, it can also impact the digestive system by binding to ACE2 receptors and causing an imbalance in gut microbiota, known as dysbiosis. Dysbiosis is linked to COVID-19 in older individuals and those with the virus. Antibiotics can cause inflammation in the intestines, which may favor the occurrence of dysbiosis in these individuals. The immune response in the gut can be influenced by bacterial metabolites and fragments, thus affecting the presentation of COVID-19. Short-chain fatty acids (SCFAs) in fiber-rich diets can protect against inflammation in the intestines. Probiotics can help improve gut dysbiosis by modulating the immune system. Personalized dietary strategies should be used in conjunction with traditional therapies. The gut microbiota can be influenced by diet and is malleable. Research could investigate the effects of COVID-19 variants on the gut microbiota and how bacteria, fungi, and phages create an environment in the stomach for COVID-19. Before and during SARS-CoV-2 infection, gut dysbiosis can affect disease progression and clinical outcomes in a self-reinforcing cycle.

The composition of the gut microbiota in COVID-19 patients during their hospitalization is associated with levels of cytokines, chemokines, and inflammation markers in their plasma. This indicates that the gut microbiota may influence the severity and course of the disease by influencing the host’s immune response. According to recent immunological studies of COVID-19 patients, the depletion of particular bacterial species was associated with increased TNF-, CXCL10, CCL2, and IL-10. This suggests that these bacteria play a role in preventing excessive inflammation. Several drugs used to treat COVID-19 may interfere with the normal functioning of the gastrointestinal (GI) tract and lead to various clinical symptoms that could further exacerbate the risk and severity of GI symptoms in COVID-19 patients, such as nausea, vomiting, gastroparesis, and gastric ulcers, through various molecular pathways and mechanisms.

The respiratory infection driven by SARS-CoV-2 affects the gastrointestinal tract microbiome, whereas respiratory infections affect the respiratory tract bacteria and immune system. However, gastrointestinal infections are often neglected. The SARS-CoV-2 microbiome, which is part of the GI tract microbiota, influences the SARS-CoV-2 immune response. Supporting this theory, depleted gut commensals, such *B. adolescentis*, *F. prausnitzii*, *E. rectale*, *R. (Blautia) obeum*, and *D. formicigenerans,* have been associated with decreased host inflammatory response in several inflammatory related disorders. *F. prausnitzii* can activate human colonic regulatory T cells that produce IL-10, which is an anti-inflammatory cytokine. *E. rectale* has been associated with reduced inflammation in Alzheimer’s disease, while *B. adolescentis* can block the activation of nuclear factor κB, a promoter of proinflammatory cytokines. Microbial-mediated immunological dysregulation is supported by the prevalence of *Ruminococcus gnavus*, *Ruminococcus torques*, *Bacteroides dorei*, and *Bacteroides vulgatus* in COVID-19 patients. Whereas *B. dorei* and *B. vulgatus* are correlated with inflammatory gut disorders, such as irritable bowel syndrome and ulcerative colitis, *R*. *gnavus* and *R. torques* have been linked to inflammatory bowel disease. It is currently unclear whether the inflammatory gut microorganisms that are more common in COVID-19 patients are actively involved in causing the disease or if they are thriving due to the absence of other beneficial gut microorganisms. The increased frequency of virus infections has allowed SARS-CoV-2 to mutate and to develop new variants (such as Alpha, Beta, Gamma, and Delta) with higher transmission rates, which can cause damage to the endothelial lining of blood vessels or disrupt the immune system. As a result, gut microbial dysbiosis may exacerbate gastrointestinal injury. This raises questions about the potential relationship between microbial diversity and health and the emergence of COVID-19 variants. The current treatment for COVID-19 with numerous variants relies on symptomatic approaches, and considering gastrointestinal problems associated with these drugs is vital. A novel potential strategy using probiotics, gut microbiota metabolites, and dietary products to enhance the gut microbiota composition in COVID-19 patients could be of great significance. Finally, the role of the lung microbiota may also be investigated. These investigations will help us understand SARS-CoV-2 variations and their effects on gut dysbiosis and probiotic regulation of the intestinal microbiota to improve COVID-19 patient health. Furthermore, the available therapeutics against COVID-19 must include modes of preventive measures apart from treatment regimes.

Traditional herbal medicines and functional foods are promising for the enhancement of innate and adaptive immune functions, and they provide extended protection against COVID-19. Many herbal formulations and functional foods have been studied for their efficacy against COVID-19, and they show potential to be developed further for safe and effective therapeutic use. Several functional foods have demonstrated anti-viral activity, an important aspect of their potential benefits against COVID-19 infection. Most functional foods have a potent immune-boosting effect, including small molecules, such as polyphenols, flavonoids, curcumin, vitamins C, E, and D, and elemental micronutrients, such as zinc. In COVID-19, omega-3 fatty acid can reduce the chance of having a SARS-CoV-2 infection, shorten the duration of symptoms, reduce the risk of renal and respiratory failure, and increase the patient’s survival rate.

Furthermore, functional foods have demonstrated health benefits in various chronic conditions, making them especially beneficial for patients with conditions including osteoporosis, obesity, diabetes, and other metabolic diseases, which make them more vulnerable to the adverse effects of SARS-CoV-2. However, the health benefits of functional foods against COVID-19 require scientific claims and optimization to establish their role in therapeutics. Additionally, there is a need for toxicological evaluation to determine the optimal dosage and safe administration of these functional foods.

## 6. Study Highlights

There is no proof of SARS-CoV-2 mutations and how they impact gut dysbiosis, but many studies have suggested a connection between COVID-19 and an imbalance in the gut flora. We investigated the effects of the various SARS-CoV-2 variants (Alpha, Beta, Gamma, Delta, and Omicron) on gut microbiota dysbiosis in COVID-19 patients. Additionally, we explored dietary interventions, such as probiotics, that could alleviate gut dysbiosis in COVID-19 patients. While traditional herbal remedies and functional foods may offer some advantages, more research is necessary to understand how they work and any drawbacks. Therefore, further research is required to fully comprehend the interactions between the virus, the host, and the microbiome and to develop more effective therapies.

## Figures and Tables

**Table 1:** Communities of bacteria seen in various areas of the respiratory and gastrointestinal systems. The asterisk (*) symbols represent the bacteria common to the respiratory and digestive systems.

Location	Microbial Diversity (Unit^−1^)	Representative Composition	Reference
Nasal cavity	1 × 10^3^	Streptococcus * *Propionibacterium* *Corynebacterium* *Moraxella*	[36]
Nasopharynx	1 × 10^3^	*Streptococcus* *Dolosigranulum* *Haemophilus*	
Oropharynx	1 × 10^6^	*Rothia* *Veillonella* *Leptotrichia* *Preuotella*	
Lung	1 × 10^2^	*Veillonella* *Preuotella* Streptococcus * *Tropheryma whipplei*	
Stomach	1 × 10^1^	*Lactobacillus* *Helicobacter* *Veillonella*	
Duodenum	1 × 10^3^	*Streptococcus* *Lactococcus* *Staphylococcus*	
Jejunum	1 × 10^4^	*Lactobacillus* *Streptococcus* *Enterococcus*	
Ileum	1 × 10^7^	*Segmented filamentous bacteria* *Enterobacteriaceae* *Bacteroides* *Clostridium*	
Colon	1 × 10^12^	*Proteobacteria* *Bacteroides* *Clostridium* *Lachospiraceae* *Prevotellaceae*	

**Table 2:** List of probiotics and their mechanisms of action in gut microbiota in patients with COVID-19. ↑ upregulate, ↓ downregulate.

Category	Source	Probiotics	Mechanism and Effects	References
Vegetables	Sauerkraut	*Lactobacillus plantarum*	Promotes the growth of beneficial probiotics, boosts the immune system, ↓ stress, ↓ risk of cancer, ↓ rate of age-related loss in bone mineral density	[1,71]
	Miso	*Aspergillus oryzae*	Starch hydrolysis, ↓ risk of cancer, heart disease	
	Kanji	Rhodotorulaglutinis	↓ cell and tissue damage	
	Cassava	*Galactomycesgeotrichum*	Starch hydrolysis	
	Pickle	*Pediococcus cerevisiae*	↓ chances of heart disease, stroke, cancer, and respiratory disease	
	Pulque	*Torulasporadelbrueckii*	Inhibits DPPH activity	
Fruits	Masau	*Saccharomyces cerevisiae*	Produces folates, which ↓ the risk of Alzheimer’s disease, cardiovascular disease	
	Cocoa	*Saccharomyces cerevisiae*, *Halimeda opuntia*	Anti-inflammatory activity, ↓ risk of irritable bowel syndrome (IBS)	
	Kombucha	*Medusomycesgisevii lindau*	Starch hydrolysis, ↓ risk of rheumatism, gout, and hemorrhoids	
	Olives	*Candida krusei*	↑ amylase and trypsin activity	
	Tepache	*Hanseniasporauvarum*	Inhibits the growth of pathogenic bacteria	
	Chili Pepper	*Hanseniasporaguilliermondii*	Prevents colonization by pathways in the human gut	
Cereals	Sourdough	*Chamaerops humilis*	Good hydrophobicity and hemolytic activity	
	Ogi	*Pichia kudriavzevii*	Helps generate hormones and lower blood pressure	
	Pozol	*Rhodotorulaminuta*	↓ secretion of Interleukin-8 (IL-8), ↓ cholesterol	
Dairy Products	Yogurt	*Lactobacillus bulgaricus*, *Streptococcus thermophilus*	↓ digestive problems and enhances gut microbiota	
	Kefir	*Kluyveromyces* spp.	Anti-inflammatory activity and anti-aging properties	
	Milk	*Lactobacillus fermentum*	↓ osteoporosis	
Plant-Based Protein	Tempeh	*Bifidobacterium*	↓ oxidative stress, ↓ cholesterol levelImproves bone health	

## Data Availability

Not applicable.
